# Effects of Exercise Interventions and Physical Activity Behavior on Cancer Related Cognitive Impairments: A Systematic Review

**DOI:** 10.1155/2016/1820954

**Published:** 2016-04-10

**Authors:** Philipp Zimmer, Freerk T. Baumann, Max Oberste, Peter Wright, Alexander Garthe, Alexander Schenk, Thomas Elter, Daniel A. Galvao, Wilhelm Bloch, Sven T. Hübner, Florian Wolf

**Affiliations:** ^1^Department of Molecular and Cellular Sport Medicine, Institute of Cardiovascular Research and Sports Medicine, German Sport University Cologne, Am Sportpark Müngersdorf 6, 50933 Cologne, Germany; ^2^Chair of Sports Medicine, Chemnitz University of Technology, Thüringer Weg 11, 09126 Chemnitz, Germany; ^3^German Center of Neurodegenerative Diseases, Arnoldstraße 18, 01307 Dresden, Germany; ^4^Department I of Internal Medicine, Center for Integrated Oncology Köln Bonn, University of Cologne, Kerpener Straße 62, 50937 Cologne, Germany; ^5^Edith Cowan University Health and Wellness Institute, Joondalup, WA 6027, Australia

## Abstract

This systematic review analyzes current data on effects of exercise interventions and physical activity behavior on objective and subjective cancer related cognitive impairments (CRCI). Out of the 19 studies which met all inclusion criteria, five RCTs investigated rodents, whereas the other 14 trials explored humans and these included six RCTs, one controlled trial, two prospective noncontrolled trials, one case series, one observational study, and three cross-sectional studies. The results from animal models revealed positive effects of exercise during and after chemotherapy or radiation on structural alterations of the central nervous system, physiological as well as neuropsychological outcomes. The overall study quality in patient studies was poor. The current data on intervention studies showed preliminary positive effects of Asian-influenced movement programs (e.g., Yoga) with benefits on self-perceived cognitive functions as well as a reduction of chronic inflammation for breast cancer patients in the aftercare. Exercise potentially contributes to the prevention and rehabilitation of CRCI. Additional RCTs with standardized neuropsychological assessments and controlling for potential confounders are needed to confirm and expand preliminary findings.

## 1. Introduction

A vast body of literature reports about a decline in subjective and objective cognitive functioning as well as structural and neurophysiological alterations of the central nervous system (CNS) after medical treatment for cancer [1]. Although the knowledge about the underlying mechanisms is sparse, results from animal studies suggest that some treatment strategies such as specific chemotherapies as well as radiation directly impair neural progenitor cells and postmitotic oligodendrocytes [2, 3]. Furthermore, markers of chronic inflammation which are frequently observed in cancer patients, such as Interleukin-1 and TNF-alpha, are associated with a decline in some cognitive domains [4]. Patients indicate limitations in various cognitive domains, for example, “executive functions,” “attention,” “memory,” and “learning” [1]. Depending on cancer type, therapy, and assessments, studies revealed a prevalence of cognitive impairments in up to 75% of cancer patients during and up to 60% after medical treatment [5, 6]. The most common terms describing this phenomenon are “chemobrain,” “chemofog,” or “post-chemotherapy cognitive impairment.” However, cognitive impairments also emerge after other types of cancer therapies, such as radiation [7], surgery [8], or hormone therapy [9]. Besides medical treatments, studies showed that cognitive abilities in cancer patients are further influenced by other factors, for example, posttraumatic stress prior to therapy [10] as well as the type of patient information on cognitive deficits as a consequence of therapy [11]. Due to its multifactorial genesis and as recommended by experts, we will use the term cancer-related cognitive impairments (CRCI) in the following [1].

In view of cancer prevention and rehabilitation, exercise programs are becoming an important part of supportive therapies in the past decades. Results from epidemiological studies showed that regular exercise and physical activity reduce cancer risk [12–14] and mortality [15]. Furthermore, exercise interventions decrease psychological and physiological disease- and treatment-specific side effects, such as fatigue [16], depression [17], lymphedema [18], and incontinence [19], leading to an increased quality of life during and after therapy [20, 21].

Regarding the reduction of side effects, the type, intensity, and duration of exercise strongly vary and comprise aerobic and resistance exercise, balance training, and Asian-influenced programs (e.g., Yoga). In general, physical activity is the sum of daily activities (gardening, movement in every-day life, etc.) and exercise (any kind of sports), whereas physical exercise is limited to any kind of sports.

In addition to all benefits named above and independently of cancer, physical activity and exercise are known to have positive effects on structural [22] adaptions of the CNS. As described for cancer, regular exercise seems to have a preventive effect regarding neurodegenerative disorders (e.g., Alzheimer and Parkinson) [23–25]. The current literature also suggests that both chronic exercise and acute exercise improve selective aspects of cognitive functioning in young and old healthy adults [26, 27]. Although there is some evidence that resistance exercise and other types of training (e.g., Yoga) have beneficial effects on cognition, most studies in this field deal with aerobic exercise programs.

Acute aerobic exercise leads to an increased expression of neurotrophic and neuroprotective factors, such as the brain-derived neurotrophic factor (BDNF) [28], the vascular endothelial growth factor (VEGF) [29], and the insulin-like growth factor (IGF1) [30] in a dose-dependent manner. Results from animal studies showed that these growth factors mainly contribute to a process called “neurogenesis” in specific brain regions, especially in the hippocampus, a highly evolutionary conserved structure which plays a key role in spatial memory and memory consolidation [31]. Interestingly, the hippocampus is degenerated by the course of neurodegenerative disorders and is further sensitive to toxic agents such as different types of chemotherapies and radiation [32–36]. Indeed, many studies revealed that exercise-induced neurogenesis is accompanied by an increased hippocampus volume as well as enhanced functioning of hippocampus-dependent cognitive abilities [22, 35]. Apart from its impact on neurotrophic factors, regular exercise contributes to establishing an anti-inflammatory environment [37, 38]. Since inflammation is a hallmark of neurodegenerative diseases [39] and is further associated with impaired cognitive functions [40], this may reflect another mechanism by which exercise counteracts such disorders. Both the neurotrophic and the anti-inflammatory effects represent acute changes in response to exercise which lead to chronic adaptions if they appear regularly.

Positive effects of exercise are not limited to hippocampus-dependent cognitive abilities. For example, improved performance of “higher,” prefrontal located cognitive skills such as executive functions (attention, response inhibition, cognitive flexibility, planning, etc.) is frequently reported after exercise [27]. However, our understanding about the underlying mechanisms of these effects is still sparse. Since the prefrontal cortex is not sensitive to neurogenesis and because of the fact that positive effects in this context are often described as “acute” [27], it would be critical to explain such improvements by an acute exercise-induced elevation of neurotrophic factors. As potential mediators of improved prefrontal cognitive function, two mechanisms are discussed. First, acute exercise is associated with the secretion of specific neurotransmitters such as dopamine (as part of the reward system) which plays an important role in prefrontal regulation [41, 42]. Second, exercise might improve the metabolic situation of neurons by providing lactate as a substrate. To date, it is well known that lactate can cross the blood-brain barrier by monocarboxylate transporters [43]. Additionally, studies showed that glucose, which is known to be the major substrate for the CNS, is frequently reduced to lactate by astrocytes before it is allocated to neurons [44].

Considering the positive influence of exercise on the CNS and the fact that cancer patients suffer from cognitive impairments, it seems plausible to bring these two areas together.

The aim of this systematic review is therefore to analyze the current literature in the context of physical activity behavior, exercise interventions, and CRCI. We also included animal studies for a more comprehensive view on potentially underlying mechanisms. Finally, we have highlighted implications and recommendations for further studies in this field.

## 2. Methods

Between February and June 2015, three independent reviewers (Philipp Zimmer, Florian Wolf, and Max Oberste) searched the databases PubMed and MEDPILOT® (Medline) for relevant literature regarding physical activity and exercise and its influence on CRCI. A study registration was not conducted. Additionally, relevant reference lists were hand-searched. According to Huang et al. [59], databases were screened by using the PICO (population, intervention, comparison, outcome) method. The following key words and MeSH terms were supplied: “tumor,” “tumour,” “neoplasms,” “metastasis,” “metastases,” “cancer,” “radiotherapy,” “radiation,” “irradiation,” “chemotherapy,” “hormonetherapy,” AND “physical activity,” “physical exercise,” “physical fitness,” “exercise,” “moving therapy,” “sports therapy,” “sports,” “training,” AND “neuropsychology,” “cognition,” “neurocognition,” “attention,” “cognition disorders,” “memory,” “problem solving,” “cognitive function,” “chemobrain,” “chemo-brain,” “chemo-fog,” “pcci,” “spatial learning,” “spatial processing.” Studies investigating CNS tumors and combined therapy studies (e.g., exercise and nutrition) were excluded since the study design does not allow unambiguous interpretation of the data. Furthermore, reviews were also excluded from analysis after screening them for potential original data. All studies which did not match the listed exclusion criteria were included. Interventional studies which did not include animals were further ranked according to the Oxford Centre for Evidence-Based Medicine (OCEBM) resulting in grades of recommendation (Table 1). This method was described as most effective by Atkins et al. [60] and was applied for other reviews in a comparable context [61, 62].

## 3. Results

**Figure 1:**
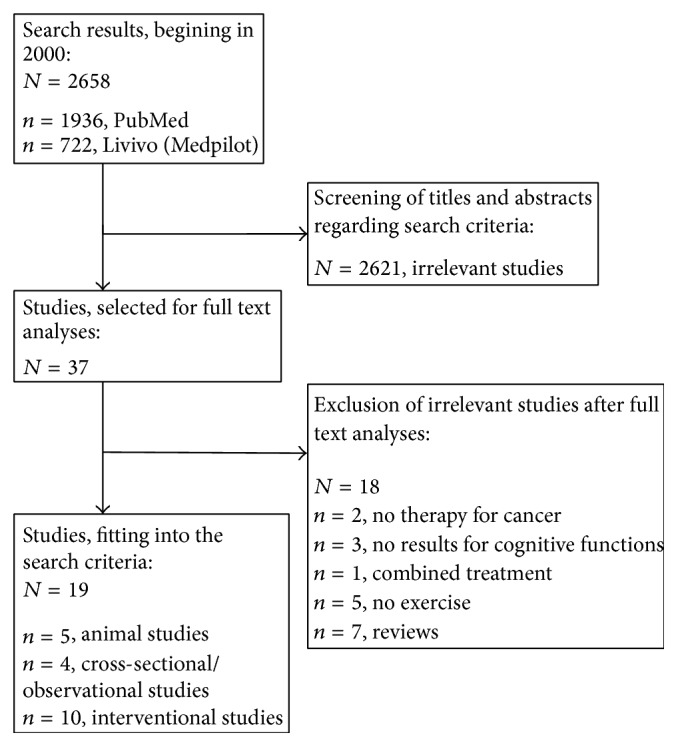
Literature search strategy.

Out of 2658 search results (PubMed: 1936, MedPilot: 722), 19 studies were chosen for further analysis. Besides five RCTs with rodents, we found 14 studies with cancer patients, including a total number of 1645 individuals. These 14 studies were further divided into six RCTs, one controlled trial, two prospective noncontrolled trials, one case series, one observational study, and three cross-sectional studies. An overview of the literature search is shown in Figure 1.

### 3.1. Animal Studies

Five RCTs with rodents, including a total number of 226 animals, investigated the impact of chemotherapy [33, 34] or radiation [32, 35, 36] in combination with exercise. As exercise interventions in all studies animals had access to a running wheel for different time periods. The cognitive tasks mainly focused on hippocampus-related cognitive functions and include variations of water maze paradigm and different memory testing. A detailed description of these studies can be found in Table 2. In general one can state that both administered chemotherapies and radiation caused a decline in cognitive functions and impairments in hippocampal neurogenesis. Independent of medical treatment, aerobic exercise improved cognitive functions in comparison to inactive control groups and led to increased levels of neurotrophic factors as well as an enhanced hippocampal neurogenesis.

### 3.2. Human Cross-Sectional and Observational Studies

Three cross-sectional studies, including 323 breast cancer patients, were conducted. However, data of Marinac et al. [47] and Hartman et al. [46] were collected in the same study population. In these studies higher levels of physical activity (which was objectively assessed by hip accelerometer and the global physical activity questionnaire) corresponded with better outcomes in several cognitive domains, for example, memory, executive functions, visual and spatial processing, attention, and speed of information processing. Effect size of physical activity (measured by accelerometer) on processing speed was higher among overweight and obese breast cancer survivors. However, these patients were three times more likely to be impaired in this cognitive domain. Hartman et al. further reported that patients in the highest tertile of physical activity (measured by questionnaire) revealed better performances in executive functions and attention, whereas patients in middle tertile of physical activity showed better result regarding the visual-spatial cognition domain. Besides exercise, sleep was also associated with the cognitive performance. Crowgey et al. [45] compared physical aerobic fitness as well as self-reported physical activity with neuropsychological assessments in breast cancer patients after chemotherapy and healthy controls. When adjusting for age, activity level, and aerobic fitness, no group differences were detected. A correlation between physical activity and cognition was only found for the visual memory domain. In addition to these cross-sectional studies, Fitzpatrick et al. [48] compared prostate and breast cancer patients receiving chemotherapy with patients in the aftercare in view of cognitive abilities and physical activity behavior. Patients undergoing chemotherapy showed impaired cognitive functions (measured by the Montreal Cognitive Assessment). After six weeks increased physical activity was associated with better performances in cognitive functions. Since this study comprises only 15 patients with different cancers and related therapies, it is difficult to interpret its findings. An overview of these studies can be found in Table 3.

### 3.3. Human Interventional Studies

An overview of all interventional studies is listed in Table 4. Six RCTs, including 1237 patients, investigated the impact of different exercise programs on CRCI. Two of the largest studies (*n* = 558) compared Yoga interventions with usual care in breast cancer patients after chemotherapy [50, 51]. While Derry et al. detected no time × group differences in self-perceived cognition after 12 weeks of Hatha Yoga, Janelsins and colleagues reported enhanced self-perceived memory function after 4 weeks of YOCAS (combination of breathing exercise, Hatha Yoga, and meditation). After a follow-up of three months, Derry et al. also described significant improvements in self-perceived cognition as well as reduced inflammation markers in the intervention group. Both studies were rated with an Oxford level of evidence as 1b.

A recent RCT of Mustian et al. [49] showed that a six-week home-based exercise program during chemotherapy in 479 nonmetastatic cancer patients consisting of aerobic walking and band resistance training results in enhanced values of self-perceived cognitive functions as well as a reduction of the inflammatory markers Interferon-*γ*, Interleukin-8, and Interleukin-1b. Furthermore, the authors described an increase of the anti-inflammatory cytokines Interleukin-6, Interleukin-10, and the soluble TNF-*α* receptor antagonist. Finally, the exercise group showed a correlation between the reduction of inflammation and changes in self-perceived cognitive function. The study was rated with an Oxford level of evidence 1b.

Oh et al. [53] investigated the influence of a ten-week Qigong intervention on self-perceived cognition, quality of life, and serum levels of the inflammation marker CRP in a heterogeneous cancer patient collective (*n* = 81). Out of the 37 patients of the intervention group, only 23 patients completed the intervention. Time × group analysis revealed improved self-perceived cognition as well as reduced CRP serum levels in participants of the intervention group. Due to the mixed study population in view of cancer type, the study was rated with 2b.

Miki et al. [52] allocated breast and prostate cancer patients in different therapy phases to an intervention group, receiving a four-week speed-feedback training, and a passive control group. The intervention consisted of two five-minute sessions per week. During these sessions, patients had to follow a pathway on a screen by adapting the number of revolutions on a bicycle ergometer. In view of its short duration and low intensity (20 Watt on a bicycle ergometer) and the additional cognitive component, this intervention should not be interpreted as classical exercise training. However, time × group analysis revealed improved prefrontal functions (assessed by the objective Frontal Assessment Battery) in the intervention group. Because of its inhomogeneous participant characteristics as well as its feasibility character, the study was also rated with 2b.

Finally, Rogers et al. [54] reported that a 12-week physical behavior educational program for breast cancer patients did not change self-perceived cognitive functions. Because of the relatively small sample size and the fact that cognitive function was only a secondary endpoint, the study was rated with 2b.

Regarding their methodological limitations due to missing of randomization or missing control groups and their small sample sizes, the studies of Knobf et al. [56], Galantino et al. [58], Reid-Arndt et al. [57], and Baumann et al. [55] were rated with grade 4.

In summary, we found three studies with a 1b level of evidence (grade of recommendation A), 3 trials with 2b (B), and four studies which were rated with 4 (C). In view of differences in exercise interventions, poor study quality, and missing pretreatment assessments, exercise recommendations to improve self-perceived cognition after chemotherapy for breast cancer patients are currently limited to Yoga based interventions to date.

## 4. Discussion

Although CRCI is a frequently observed side-effect in cancer patients and physical activity and exercise interventions are known to have beneficial effects on cognitive functions, only very few human studies with predominately methodological limitations were conducted so far. Furthermore, results from cross-sectional studies suggest that elevated levels of physical activity are associated with fewer declines in cognitive function in cancer patients. Furthermore, Asian-related movement interventions seem to have a positive influence on self-perceived cognitive abilities and may reduce systemic inflammation in the aftercare. The major limitations of all interventional exercise studies are the designs (missing randomization or complete absents of control groups), missing pretherapy data, and the usage of heterogeneous neuropsychological assessments (mainly varying questionnaires detecting self-perceived cognition). As a result, there are no current specific exercise recommendations to counteract CRCI. The named limitations should not be seen as criticism in general, since this research field is quite new and the cited studies have pioneer character.

The findings of the described animal studies clearly indicate that different cancer therapies, such as chemotherapy and radiation, are strongly associated with structural and functional changes of the CNS. All of these studies revealed that exercise is a promising method to counteract this negative therapy-dependent development. At present results from animal studies are difficult to translate to humans in the context of exercise and CRCI for the following reasons:

- The cited animal studies used endurance exercise interventions which seem to be plausible because endurance exercise is the most frequently investigated type of exercise in cognition studies. However, only one of the human studies with a low explanatory power (no control group, only subjective cognition assessment) [56] used a comparable aerobic endurance exercise protocol which is even more astonishing since recommendations of experts suggest moderate-to-vigorous endurance exercise for brain health [63].
- With only a few exceptions, animal studies focused on hippocampus-dependent cognitive functions (e.g., spatial memory). Although a translation from the rodent to the human brain is difficult in many cases, spatial memory seems to be a hippocampus-related function in humans as well. This may be reasoned by the fact that the hippocampus is an evolutionary ancient and highly conserved structure [64]. However, CRCI also affects “higher” cognitive functions which are predominately located in the prefrontal cortex. In contrast to the hippocampus, the prefrontal cortices of animals and humans are incommensurable structures. The prefrontal cortex has also been described as the “human” part of the brain. While the prefrontal cortex represents about 29% of the humans' cortex volume, this number is broadly smaller in animals (e.g., 3.5% in cats and 11.5% in macaques) [65]. As mentioned above, the translation of results from animal studies, especially in view of prefrontal located “higher” cognitive functions, can only be made with caution.

Nevertheless, animal studies gave first hints about underlying mechanisms of exercise-induced improvements of brain function. As stated above, neurogenesis in the hippocampus is strongly affected by both CRCI and exercise. Therefore, hippocampus-dependent cognitive functions represent a promising target for further research in humans. When planning such studies with regard to the assessment of cognitive functions, one has to consider that neurogenesis and following functional embedding of the new neurons are a process which takes at least four to six weeks [66, 67]. Shorter measurement intervals might lead to confusing results. As such, the inclusion of follow-up measurements would be ideal. Against the background of exercise-induced neurogenesis, neuropsychological assessments should focus on hippocampus functioning and additionally include general assessments which are advised by the international cognition and cancer task force [68]. Regarding the applied exercise regime, following studies are recommended to use different types of endurance/aerobic exercise and maybe also varying intensities. From a biological point of view this could be argued by the fact that endurance exercise is known to stimulate the expression of neurotrophic factors, such as BDNF and VEGF in an intensity-dependent manner [69]. However, first studies showed that resistance exercise also increases some of these agents [70]. Since the exercise-driven secretion of neurotrophic factors is a typical short-term effect, the question whether the assessment of these factors should take place at the same measurement time points as the cognition testing arises. We hypothesize that it may be of greater interest to investigate differences of short-term courses of these factors, for example, before and after the first and the last exercise sessions in an interventional study comparing different (endurance) exercise intensities. Thereby, further studies may be able to determine if the peak or a certain threshold of neuronal growth factor secretion is pivotal for neurogenesis and if the expression of those factors changes during the time course of the intervention.

Recent research suggested that exercise alone might not be sufficient to induce a long-lasting, functional neurogenesis [71, 72]. Fabel et al. [73] revealed that exercise enhanced the proliferation of neuronal progenitor cells in the hippocampus, thereby creating a “neurogenic potential.” A majority of these new born cells did only reach functionality when exercise was combined with cognitive training. Similar results were reported for humans by Fabre and colleagues [74]. Therefore, the combination of aerobic exercise with cognitive training depicts a promising strategy to improve hippocampus-dependent cognitive functions in CRCI.

Among typical prefrontal cortex-dependent cognitive abilities, executive functions were reported to be most sensitive to exercise intervention in healthy adults [72]. Interestingly, executive functions are frequently impaired in patients suffering from CRCI [75]. To date, there is no generally accepted standard definition of executive function. It has become common practice to define executive functioning by enumerating subcomponents such as task flexibility, response inhibition, reasoning, problem solving, selective attention, and planning [76]. Since neurogenesis seems to be limited to only very few brain regions (e.g., hippocampus and olfactory epithelium) it remains at least questionable if new born neurons take place and function in other regions of the CNS. Thus, an exercise-induced increased performance in executive functions might be driven by other mechanisms. Indeed, exercise was reported to elevate levels of neurotransmitters (e.g., dopamine), which are associated with prefrontal cortex functions [41, 42]. In addition, first studies showed that an acute increase in lactate may ameliorate neuronal function. Lactate is known to cross the blood-brain barrier and is used as energy substrate by neurons. Furthermore, exercise induces an increase in synaptic plasticity and reduces chronic inflammation [37, 38]. Since chronic inflammation is commonly observed in cancer patients and is further associated with cognitive performance [40], pro- and anti-inflammatory cytokines will be an interesting target for studies dealing with exercise and CRCI. Finally, results from neurophysiological investigations suggested that single bouts of exercise lead to a reduced activation of the prefrontal cortex which was thought to be some kind of “relaxing.” This “relaxing” phase during exercise was further discussed to improve cognitive functions after cessation of exercise [77].

It can be summarized that prefrontal cortex functions, especially selective aspects of executive functions, may change after exercise. Besides adequate testing of executive functions, at least some of the potentially underlying mechanisms should be considered when planning exercise interventions in the context of CRCI.

Besides aerobic exercise, Asian-influenced movement programs display a promising behavioral approach to counteract CRCI. The results and the explanatory power of these studies are hard to compare due to different assessments which were used to determine subjective cognitive impairments (Table 4). In addition to the cited research in the context of CRCI, Yoga has been shown to improve symptoms in patients suffering from other CNS disorders [78]. However, the underlying mechanisms may differ from those of aerobic exercise since Asian-influenced exercise programs are more related to improvements in mood, motivation, and mindfulness [79]. An interesting common effect of both types of interventions is amelioration in sleep [46, 80]. Since better sleep is associated with increased cognitive performances [46] it should be considered as a potential mediator. A comparison regarding the effects of aerobic exercise and Yoga-like interventions is not appropriate at this time due to underpowered studies and strongly varying endpoints (objective and subjective) measurements of CRCI. Further research may include a combination of both.

As a trap door for all exercise interventions in the context of brain function, the study design represents a general problem. Since the performance in objective and subjective neuropsychological tests is affected by mood, motivation, and other factors [81, 82], future studies should randomize patients in exercise groups, placebo control groups, and if possible a passive control group to estimate potential placebo effects. In many studies which investigate the impact of exercise interventions on cognitive functions, control groups did not receive a comparable social support (missing placebo control group) or even were cognitive exhausted by tasks such as book-reading. These nuisances in study designs may lead to overestimated effects of exercise.

Apart from study design and the hypothesis-driven objective and subjective neuropsychological as well as neurobiological assessments, potential confounding factors should always be included when planning research on exercise interventions in the context of CRCI. These confounding factors include intelligence quotient, age, posttraumatic stress prior to therapy, sleep, activity behavior, depression symptoms, and fatigue. It is not worth stating that study collectives should consist of similar cancer types receiving identical therapy protocols and patient information about potential cognitive impairments. Thus, evidence-based recommendations for exercise programs as part of supportive therapy can be further developed regarding the treatment of CRCI.

Finally researchers have to determine how much assessment is acceptable for patients. In particular the outcomes of neuropsychological assessments depend on motivational aspects [82]. Therefore, cognitive functions should be tested with a specific aim (e.g., hippocampal function) and may be executed in a randomized fashion. Executing too many neuropsychological tests, even if applied in a counterbalanced manner, affects test power since the mean motivation among participants decreases and the mean cognitive load when performing a certain task increases [82].

A last aspect which should be taken into account when discussing treatment strategies of CRCI is the cognitive reserve theory (for review [83]). This theory hypothesized that people with higher cognitive functions need longer time to reveal clinical significant cognitive impairments compared to people with lower cognitive abilities. Regular physical activity and different types of exercise may increase the individual cognitive reserve. This mechanism could contribute to a delay in the incidence of CRCI in physical active patients.

At present some promising trials are underway but are not published yet. To give two examples, Matthews et al. [84] compare the impact of a five-month home-based aerobic exercise intervention to a standard educational behavior strategy program regarding cognitive functions in 64 cancer patients and Campbell [85] conducts a study comparing aerobic exercise with usual care in breast cancer patients. Besides subjective and objective neuropsychological assessments, the latter trial also includes fMRI analysis.

The results of the present review should be considered within the context of its limitations. Study selection and ranking were performed by three reviewers in order to minimize subjectivity. However, selection bias can never be ruled out completely. Furthermore, the ranking according to the Oxford levels of evidence was aggravated by the accessibility to adverse events, raw data, and confidence intervals. Therefore, over- or underestimating of studies cannot be entirely eliminated.

## 5. Conclusion

Results from animal studies clearly indicate that exercise interventions represent an effective method to counteract CRCI on the structural and functional level in rodents, especially regarding hippocampus-dependent functions. However, CRCI-associated cognitive impairments in humans are not limited to hippocampus-dependent functions and also affect other brain regions, such as the prefrontal cortex, which correspond with “higher” cognitive functions. Since the prefrontal cortices of humans and rodents are hard to compare, results from animal studies should only be carefully translated to humans. In humans, more RCTs, using appropriate control groups, standardized neuropsychological assessments (according to the recommendations of the Cancer and Cognition Task Force), and patient information about cognitive side effects are required. Furthermore, recording of potential confounders, such as posttraumatic stress, depressions, fatigue, and age, is necessary. Finally, one should always scrutinize if the scheduled exercise intervention is associated with improvements in the assessed cognitive domains when planning an interventional study.

## Figures and Tables

**Table 1:** Oxford levels of evidence and grades of recommendation.

Level	Content	Grade of recommendation
1a	Systematic reviews with homogeneity in the case of randomized controlled trials	A
1b	Individual randomized controlled trials (with narrow confidence interval)	

2a	Systematic reviews with homogeneity of cohort studies	B
2b	Individual cohort study (including low-quality, randomized controlled trials)	
3a	Systematic reviews with homogeneity of case-control studies	
3b	Individual case-control study	

4	Case series (and poor-quality cohort and case-control studies)	C

5	Expert opinion without explicit critical appraisal	D

**Table 2:** Exercise interventions in rodents.

Reference	*N*	Study design	Study population	Treatment	Type of exercise	Duration	Parameters
Chemotherapy							
Winocur et al., 2014 [33]	38 9 MTX/5FU Run 10 MTX/5FU 9 CG Run 9 CG	2 × 2 RCT	f, Long-Evans rats	37.8 mg/kg MTX + 50 mg/kg 5FU or salt solution	Access to a running wheel	11 weeks	SM (↑) CM (→) NMTS (↑) DNMTS (↑) Hippocampal neurogenesis (↑)
Fardell et al., 2012 [34]	28 7 5FU/OX Run 7 5FU/OX 7 CG Run 7 CG	2 × 2 RCT	m, hooded Wistar rats	75 mg/kg FU + 8 mg/kg OX or salt solution	Access to a running wheel overnight	6 weeks	NOR (↑) MWM (↑)

Cranial radiation							
Ji et al., 2014 [32]	104 16 20 Gy Run 16 20 Gy 16 CG Run 16 CG	2 × 2 RCT	Sprague-Dawley rats	20 Gy or sham radiation	30 min access to a running wheel in the morning and evening	5x/week over 3 weeks	Open-field test (→) MWM (↑) BDNF (↑) Hippocampal neurogenesis (↑)
Wong-Goodrich et al., 2010 [35]	40 10 5 Gy Run 10 5 Gy 10 CG Run 10 CG	2 × 2 RCT	f, C57BL/6 mice	5 Gy or sham radiation	Access to a running wheel 8/12 hours per day	16 weeks	Barnes Maze (↑) Hippocampal neurogenesis (↑) Growth factor expression (↑)
Naylor et al., 2008 [36]	16 4 6 Gy Run 4 6 Gy 4 CG Run 4 CG	2 × 2 RCT	C57BL/6 mice	6 Gy or sham radiation	Access to a running wheel	4 weeks	Open-field test (↑) Hippocampal neurogenesis (↑)

5FU: 5-Fluorouracil; OX: Oxaliplatin; CG: control group; Gy: Gray; RCT: Randomized Controlled Trial; m: male; f: female; NOR: Novel Object Recognition; MWM: Morris Water Maze; SM: spatial memory; CM: Cued Memory; NMTS: Non-Matching to Sample Task, DNMTS: Delayed Non-Matching to Sample task.

**Table 3:** Human cross-sectional and observational studies.

Reference	*N*	Study design	Study population	Status of therapy	Duration	Parameters	Correlations
Crowgey et al., 2014 [45]	51 37 chemotherapy 14 healthy	Cross-sectional study	Breast cancer	After chemotherapy, during hormone therapy		(1) Self-reported physical activity (Leisure Score Index) (2) Cardiovascular fitness (VO_2_ peak) (3) Central nervous system vital signs software	LSI, visual memory

Hartman et al., 2015 [46]	136	Cross-sectional study	Breast cancer	After chemotherapy, during hormone therapy		(1) Physical activity (2) Cognitive function (a) Memory (b) Executive function (c) Visual areal processing (d) Attention (e) Information processing	Physical activity, executive function Physical activity, attention

Marinac et al., 2015 [47]	136	Cross-sectional study	Breast cancer	After chemotherapy, during hormone therapy	1 week (time of activity tracking)	(1) Physical activity (low, moderate, inactive) (2) Cognitive function (a) Information processing (b) Memory (c) Executive function	MKA, information processing

Fitzpatrick et al., 2012 [48]	15 8 during chemotherapy 7 after chemotherapy	Cohort study	Prostate and breast cancer	During and after chemotherapy	6 weeks	(1) Cognitive function (a) Montreal Cognitive Assessment (b) Healthy brain questionnaire (2) MET	MoCA, MET

MPA: Moderate Physical Activity; PA: physical activity; LSI: Leisure Score Index; MET: Metabolic Equivalent of Task; fMRI: functional Magnetic Resonance Imaging; MoCA: Montreal Cognitive Assessment.

**Table 4:** Human interventional studies.

Reference	*N*	Study design	Study population	Status of therapy	Type of exercise	Duration	Frequency	Parameters	LOE	Level of recommendation
Mustian et al., 2015 [49]	479	RCT	84% breast cancer 94% female Nonmetastatic	During chemotherapy	Home based walking and resistance band training	6 weeks	—	FACT-Cog (↑) Inflammatory markers (↓) Anti-inflammatory markers (↑)	1b	A

Derry et al., 2015 [50]	200 100 IG 100 CG	RCT	Breast cancer	After chemotherapy, during hormone therapy	Hatha Yoga	12 weeks	2x/week 90 min	Self-reported cognitive function (BCPT) (→ after intervention, ↑ after 3-month follow-up) and inflammation (→ after intervention, ↑ after 3-month follow-up)	1b	A

Janelsins et al., 2012 [51]	358	RCT	75% breast cancer 96% female	2–24 months after different adjuvant therapies	Breathing exercise, Yoga, and meditation	4 weeks	2x/week 75 min	Difficulty in remembering things (Modified MD Anderson Symptom Inventory ↑)	1b	A

Miki et al., 2014 [52]	78 38 IG 40 CG	RCT	Breast cancer, age of participants > 65 years	Therapy for cancer with varying treatments	Speed-feedback therapy on a bicycle ergometer	4 weeks	1x/week 5 min	Frontal assessment battery (↑)	2b	B

Oh et al., 2012 [53]	81 37 IG 44 CG	RCT	Breast cancer, lung cancer, prostate cancer, colorectal carcinoma, and stomach cancer	During and after chemotherapy	Medical Qigong	10 weeks	2x/week 90 min	Self-reported cognitive function: EORTC QLQ-C30 (↑), FACT-Cog (↑), and CRP (↑)	2b	B

Rogers et al., 2009 [54]	41 21 IG 20 CG	RCT	Breast cancer	>3 months after chemotherapy, during hormone therapy	Physical activity behavior change program	12 weeks	—	FACT-Cog (→)	2b	B

Baumann et al., 2011 [55]	17 9 IG 8 CG	Controlled trial	Breast cancer	During chemotherapy	Strength training	12 weeks	2x/week 60 min	Neuropsychological tests: verbal memory MEMO (↑), working memory WIT (→), and attention d2-test (↑)	4	C

Knobf et al., 2014 [56]	26	Uncontrolled trial	Breast cancer	<36 months after chemotherapy	Progressive aerobic endurance training on a treadmill (60–75% Hfmax)	6 months	3x/week 10–45 min	BCPT (↓ forgetfulness, → concentration)	4	C

Reid-Arndt et al., 2012 [57]	23	Uncontrolled trial	Breast cancer, ovarian cancer, endometrial cancer, non-Hodgkin lymphoma, and chronic lymphocytic leukemia	>12 months after chemotherapy	Tai chi	10 weeks	2x/week 60 min	Neuropsychological tests: memory (↑ for some patients), executive function (→), speech (→), and attention (↑ for some patients) Subjective problems: multiple abilities self-reported questionnaire (↑ verbal memory, visual memory)	4	C

Galantino et al., 2012 [58]	4	Case series	Breast cancer	Before, during, and after chemotherapy	Iyengar Yoga	12 weeks	1-2x/week 70 min	Perceived cognition questionnaire (↑/→/↑/→), CogState (↑ speed, ↓/→ accuracy, ↑ errors)	4	C

IG: intervention group; CG: control group; BCPT: Breast Cancer Prevention Trial; EORTC QLQ-C30: European Organization for Research and Treatment of Cancer, Quality of Life Questionnaire; FACT-Cog: Functional Assessment of Cancer Therapy-Cognitive Function; CRP: C-Reactive Protein; WIT: Wilde Intelligence Subtest.

## References

[1] Wefel J. S., Kesler S. R., Noll K. R., Schagen S. B. (2015). Clinical characteristics, pathophysiology, and management of noncentral nervous system cancer-related cognitive impairment in adults. *CA Cancer Journal for Clinicians*.

[2] Han R., Yang Y. M., Dietrich J., Luebke A., Mayer-Pröschel M., Noble M. (2008). Systemic 5-fluorouracil treatment causes a syndrome of delayed myelin destruction in the central nervous system. *Journal of Biology*.

[3] Dietrich J., Han R., Yang Y., Mayer-Pröschel M., Noble M. (2006). CNS progenitor cells and oligodendrocytes are targets of chemotherapeutic agents in vitro and in vivo. *Journal of Biology*.

[4] Patel S. K., Wong A. L., Wong F. L. (2015). Inflammatory biomarkers, comorbidity, and neurocognition in women with newly diagnosed breast cancer. *Journal of the National Cancer Institute*.

[5] Janelsins M. C., Kesler S. R., Ahles T. A., Morrow G. R. (2014). Prevalence, mechanisms, and management of cancer-related cognitive impairment. *International Review of Psychiatry*.

[6] Wefel J. S., Saleeba A. K., Buzdar A. U., Meyers C. A. (2010). Acute and late onset cognitive dysfunction associated with chemotherapy in women with breast cancer. *Cancer*.

[7] Shibayama O., Yoshiuchi K., Inagaki M. (2014). Association between adjuvant regional radiotherapy and cognitive function in breast cancer patients treated with conservation therapy. *Cancer Medicine*.

[8] Debess J., Riis J. Ø., Pedersen L., Ewertz M. (2009). Cognitive function and quality of life after surgery for early breast cancer in North Jutland, Denmark. *Acta Oncologica*.

[9] Schilder C. M., Seynaeve C., Beex L. V. (2010). Effects of tamoxifen and exemestane on cognitive functioning of postmenopausal patients with breast cancer: results from the neuropsychological side study of the tamoxifen and exemestane adjuvant multinational trial. *Journal of Clinical Oncology*.

[10] Hermelink K., Voigt V., Kaste J. (2015). Elucidating pretreatment cognitive impairment in breast cancer patients: the impact of cancer-related post-traumatic stress. *Journal of the National Cancer Institute*.

[11] Schagen S. B., Das E., Vermeulen I. (2012). Information about chemotherapy-associated cognitive problems contributes to cognitive problems in cancer patients. *Psycho-Oncology*.

[12] Zhong S., Ma T., Chen L. (2015). Physical activity and risk of lung cancer: a meta-analysis. *Clinical Journal of Sport Medicine*.

[13] Behrens G., Jochem C., Keimling M., Ricci C., Schmid D., Leitzmann M. F. (2014). The association between physical activity and gastroesophageal cancer: systematic review and meta-analysis. *European Journal of Epidemiology*.

[14] Wu Y., Zhang D., Kang S. (2013). Physical activity and risk of breast cancer: a meta-analysis of prospective studies. *Breast Cancer Research and Treatment*.

[15] Schmid D., Leitzmann M. F. (2014). Association between physical activity and mortality among breast cancer and colorectal cancer survivors: a systematic review and meta-analysis. *Annals of Oncology*.

[16] Cramp F., Byron-Daniel J. (2012). Exercise for the management of cancer-related fatigue in adults. *Cochrane Database of Systematic Reviews*.

[17] Tomlinson D., Diorio C., Beyene J., Sung L. (2014). Effect of exercise on cancer-related fatigue: a meta-analysis. *American Journal of Physical Medicine and Rehabilitation*.

[18] Schmitz K. H., Ahmed R. L., Troxel A. (2009). Weight lifting in women with breast-cancer-related lymphedema. *New England Journal of Medicine*.

[19] Zhang A. Y., Bodner D. R., Fu A. Z. (2015). Effects of patient centered interventions on persistent urinary incontinence after prostate cancer treatment: a randomized, controlled trial. *The Journal of Urology*.

[20] Mishra S. I., Scherer R. W., Geigle P. M. (2012). Exercise interventions on health-related quality of life for cancer survivors. *Cochrane Database of Systematic Reviews*.

[21] Mishra S. I., Scherer R. W., Snyder C., Geigle P. M., Berlanstein D. R., Topaloglu O. (2012). Exercise interventions on health-related quality of life for people with cancer during active treatment. *Cochrane Database of Systematic Reviews*.

[22] Erickson K. I., Leckie R. L., Weinstein A. M. (2014). Physical activity, fitness, and gray matter volume. *Neurobiology of Aging*.

[23] Mattson M. P. (2015). Lifelong brain health is a lifelong challenge: from evolutionary principles to empirical evidence. *Ageing Research Reviews*.

[24] Paillard T., Rolland Y., de Barreto P. S. (2015). Protective effects of physical exercise in Alzheimer’s disease and Parkinson’s disease: a narrative review. *Journal of Clinical Neurology*.

[25] Xu Q., Park Y., Huang X. (2010). Physical activities and future risk of Parkinson disease. *Neurology*.

[26] Lees C., Hopkins J. (2013). Effect of aerobic exercise on cognition, academic achievement, and psychosocial function in children: a systematic review of randomized control trials. *Preventing Chronic Disease*.

[27] Chang Y. K., Labban J. D., Gapin J. I., Etnier J. L. (2012). The effects of acute exercise on cognitive performance: a meta-analysis. *Brain Research*.

[28] Szuhany K. L., Bugatti M., Otto M. W. (2015). A meta-analytic review of the effects of exercise on brain-derived neurotrophic factor. *Journal of Psychiatric Research*.

[29] De Almodovar C. R., Lambrechts D., Mazzone M., Carmeliet P. (2009). Role and therapeutic potential of VEGF in the nervous system. *Physiological Reviews*.

[30] Skriver K., Roig M., Lundbye-Jensen J. (2014). Acute exercise improves motor memory: exploring potential biomarkers. *Neurobiology of Learning and Memory*.

[31] Rolls E. T. (1991). Functions of the primate hippocampus in spatial and nonspatial memory. *Hippocampus*.

[32] Ji J.-F., Ji S.-J., Sun R. (2014). Forced running exercise attenuates hippocampal neurogenesis impairment and the neurocognitive deficits induced by whole-brain irradiation via the BDNF-mediated pathway. *Biochemical and Biophysical Research Communications*.

[33] Winocur G., Wojtowicz J. M., Huang J., Tannock I. F. (2014). Physical exercise prevents suppression of hippocampal neurogenesis and reduces cognitive impairment in chemotherapy-treated rats. *Psychopharmacology*.

[34] Fardell J. E., Vardy J., Shah J. D., Johnston I. N. (2012). Cognitive impairments caused by oxaliplatin and 5-fluorouracil chemotherapy are ameliorated by physical activity. *Psychopharmacology*.

[35] Wong-Goodrich S. J. E., Pfau M. L., Flores C. T., Fraser J. A., Williams C. L., Jones L. W. (2010). Voluntary running prevents progressive memory decline and increases adult hippocampal neurogenesis and growth factor expression after whole-brain irradiation. *Cancer Research*.

[36] Naylor A. S., Bull C., Nilsson M. K. L. (2008). Voluntary running rescues adult hippocampal neurogenesis after irradiation of the young mouse brain. *Proceedings of the National Academy of Sciences of the United States of America*.

[37] Hayashino Y., Jackson J. L., Hirata T. (2014). Effects of exercise on C-reactive protein, inflammatory cytokine and adipokine in patients with type 2 diabetes: a meta-analysis of randomized controlled trials. *Metabolism: Clinical and Experimental*.

[38] Walsh N. P., Gleeson M., Shephard R. J. (2011). Position statement. Part one. Immune function and exercise. *Exercise Immunology Review*.

[39] Fakhoury M. (2015). Role of immunity and inflammation in the pathophysiology of neurodegenerative diseases. *Neurodegenerative Diseases*.

[40] Windham B. G., Simpson B. N., Lirette S. (2014). Associations between inflammation and cognitive function in African Americans and European Americans. *Journal of the American Geriatrics Society*.

[41] Baek D., Lee C., Baek S. (2014). Effect of treadmill exercise on social interaction and tyrosine hydroxylase expression in the attention-deficit/hyperactivity disorder rats. *Journal of Exercise Rehabilitation*.

[42] O'Dell S. J., Gross N. B., Fricks A. N., Casiano B. D., Nguyen T. B., Marshall J. F. (2007). Running wheel exercise enhances recovery from nigrostriatal dopamine injury without inducing neuroprotection. *Neuroscience*.

[43] Vijay N., Morris M. E. (2014). Role of monocarboxylate transporters in drug delivery to the brain. *Current Pharmaceutical Design*.

[44] Pellerin L., Magistretti P. J. (2012). Sweet sixteen for ANLS. *Journal of Cerebral Blood Flow and Metabolism*.

[45] Crowgey T., Peters K. B., Hornsby W. E. (2014). Relationship between exercise behavior, cardiorespiratory fitness, and cognitive function in early breast cancer patients treated with doxorubicin-containing chemotherapy: a pilot study. *Applied Physiology, Nutrition and Metabolism*.

[46] Hartman S. J., Marinac C. R., Natarajan L., Patterson R. E. (2015). Lifestyle factors associated with cognitive functioning in breast cancer survivors. *Psycho-Oncology*.

[47] Marinac C. R., Godbole S., Kerr J., Natarajan L., Patterson R. E., Hartman S. J. (2015). Objectively measured physical activity and cognitive functioning in breast cancer survivors. *Journal of Cancer Survivorship*.

[48] Fitzpatrick T. R., Edgar L., Holcroft C. (2012). Assessing the relationship between physical fitness activities, cognitive health, and quality of life among older cancer survivors. *Journal of Psychosocial Oncology*.

[49] Mustian K. M., Janelsins M. C., Peppone L. J. (2015). EXCAP exercise effects on cognitive impairment and inflammation: a URCC NCORP RCT in 479 cancer patients. *Journal of Clinical Oncology*.

[50] Derry H. M., Jaremka L. M., Bennett J. M. (2015). Yoga and self-reported cognitive problems in breast cancer survivors: a randomized controlled trial. *Psycho-Oncology*.

[51] Janelsins M. C., Peppone L. J., Heckler C. E. (2012). YOCAS yoga, fatigue, memory difficulty, and quality of life: results from a URCC CCOP randomized, controlled clinical trial among 358 cancer survivors. *ASCO Meeting Abstracts*.

[52] Miki E., Kataoka T., Okamura H. (2014). Feasibility and efficacy of speed-feedback therapy with a bicycle ergometer on cognitive function in elderly cancer patients in Japan. *Psycho-Oncology*.

[53] Oh B., Butow P. N., Mullan B. A. (2012). Effect of medical Qigong on cognitive function, quality of life, and a biomarker of inflammation in cancer patients: a randomized controlled trial. *Supportive Care in Cancer*.

[54] Rogers L. Q., Hopkins-Price P., Vicari S. (2009). A randomized trial to increase physical activity in breast cancer survivors. *Medicine and Science in Sports and Exercise*.

[55] Baumann F. T., Drosselmeyer N., Leskaroski A. (2011). 12-Week resistance training with breast cancer patients during chemotherapy: effects on cognitive abilities. *Breast Care*.

[56] Knobf M. T., Thompson A. S., Fennie K., Erdos D. (2014). The effect of a community-based exercise intervention on symptoms and quality of Life. *Cancer Nursing*.

[57] Reid-Arndt S. A., Matsuda S., Cox C. R. (2012). Tai Chi effects on neuropsychological, emotional, and physical functioning following cancer treatment: a pilot study. *Complementary Therapies in Clinical Practice*.

[58] Galantino M. L., Greene L., Daniels L., Dooley B., Muscatello L., O'Donnell L. (2012). Longitudinal impact of yoga on chemotherapy-related cognitive impairment and quality of life in women with early stage breast cancer: a case series. *Explore: The Journal of Science and Healing*.

[59] Huang X., Lin J., Demner-Fushman D. (2006). Evaluation of PICO as a knowledge representation for clinical questions. *AMIA Annual Symposium Proceedings*.

[60] Atkins D., Eccles M., Flottorp S. (2004). Systems for grading the quality of evidence and the strength of recommendations I: critical appraisal of existing approaches The GRADE Working Group. *BMC Health Services Research*.

[61] Streckmann F., Zopf E. M., Lehmann H. C. (2014). Exercise intervention studies in patients with peripheral neuropathy: a systematic review. *Sports Medicine*.

[62] Baumann F. T., Zopf E. M., Bloch W. (2012). Clinical exercise interventions in prostate cancer patients—a systematic review of randomized controlled trials. *Supportive Care in Cancer*.

[63] Ahlskog J. E., Geda Y. E., Graff-Radford N. R., Petersen R. C. (2011). Physical exercise as a preventive or disease-modifying treatment of dementia and brain aging. *Mayo Clinic Proceedings*.

[64] West M. J. (1990). Stereological studies of the hippocampus: a comparison of the hippocampal subdivisions of diverse species including hedgehogs, laboratory rodents, wild mice and men. *Progress in Brain Research*.

[65] Karnath H., Thier P. (2012). *Kognitive Neurowissenschaften*.

[66] Ge S., Sailor K. A., Ming G.-L., Song H. (2008). Synaptic integration and plasticity of new neurons in the adult hippocampus. *Journal of Physiology*.

[67] Zhao C., Deng W., Gage F. H. (2008). Mechanisms and functional implications of adult neurogenesis. *Cell*.

[68] Wefel J. S., Vardy J., Ahles T., Schagen S. B. (2011). International Cognition and Cancer Task Force recommendations to harmonise studies of cognitive function in patients with cancer. *The Lancet Oncology*.

[69] Ferris L. T., Williams J. S., Shen C.-L. (2007). The effect of acute exercise on serum brain-derived neurotrophic factor levels and cognitive function. *Medicine and Science in Sports and Exercise*.

[70] Yarrow J. F., White L. J., McCoy S. C., Borst S. E. (2010). Training augments resistance exercise induced elevation of circulating brain derived neurotrophic factor (BDNF). *Neuroscience Letters*.

[71] Karr J. E., Areshenkoff C. N., Rast P., Garcia-Barrera M. A. (2014). An empirical comparison of the therapeutic benefits of physical exercise and cognitive training on the executive functions of older adults: a meta-analysis of controlled trials. *Neuropsychology*.

[72] Hötting K., Röder B. (2013). Beneficial effects of physical exercise on neuroplasticity and cognition. *Neuroscience and Biobehavioral Reviews*.

[73] Fabel K., Wolf S. A., Ehninger D., Babu H., Leal-Galicia P., Kempermann G. (2009). Additive effects of physical exercise and environmental enrichment on adult hippocampal neurogenesis in mice. *Frontiers in Neuroscience*.

[74] Fabre C., Chamari K., Mucci P., Massé-Biron J., Préfaut C. (2002). Improvement of cognitive function by mental and/or individualized aerobic training in healthy elderly subjects. *International Journal of Sports Medicine*.

[75] Lindner O. C., Phillips B., McCabe M. G. (2014). A meta-analysis of cognitive impairment following adult cancer chemotherapy. *Neuropsychology*.

[76] Lezak M. D. (2004). *Neuropsychological Assessment*.

[77] Vogt T., Herpers R., Scherfgen D., Strüder H. K., Schneider S. (2015). Neuroelectric adaptations to cognitive processing in virtual environments: an exercise-related approach. *Experimental Brain Research*.

[78] Wu E., Barnes D. E., Ackerman S. L., Lee J., Chesney M., Mehling W. E. (2015). Preventing loss of independence through exercise (PLIÉ): qualitative analysis of a clinical trial in older adults with dementia. *Aging and Mental Health*.

[79] Mackenzie M. J., Carlson L. E., Ekkekakis P., Paskevich D. M., Culos-Reed S. N. (2013). Affect and mindfulness as predictors of change in mood disturbance, stress symptoms, and quality of life in a community-based yoga program for cancer survivors. *Evidence-Based Complementary and Alternative Medicine*.

[80] Payne J. K., Held J., Thorpe J., Shaw H. (2008). Effect of exercise on biomarkers, fatigue, sleep disturbances, and depressive symptoms in older women with breast cancer receiving hormonal therapy. *Oncology Nursing Forum*.

[81] Lagner P., Kliegel M., Phillips L. H. (2015). Mood effects on memory and executive control in a real-life situation. *Cognition and Emotion*.

[82] Foussias G., Siddiqui I., Fervaha G. (2015). Motivated to do well: an examination of the relationships between motivation, effort, and cognitive performance in schizophrenia. *Schizophrenia Research*.

[83] Stern Y. (2009). Cognitive reserve. *Neuropsychologia*.

[84] Matthews C. E. (2008). *The Activity Intervention for Chemobrain (TACTIC)*.

[85] Campbell K. Can Exercise Improve Cancer Associated Cognitive Dysfunction? (chemobrain). http://clinicaltrials.gov/ct2/show/NCT01296893.

